# Prenatal origin of childhood AML occurs less frequently than in childhood ALL

**DOI:** 10.1186/1471-2407-6-100

**Published:** 2006-04-21

**Authors:** Tatiana Burjanivova, Jozef Madzo, Katerina Muzikova, Claus Meyer, Bjoern Schneider, Felix Votava, Rolf Marschalek, Jan Stary, Jan Trka, Jan Zuna

**Affiliations:** 1 CLIP – Childhood Leukaemia Investigation Prague, Czech Republic; 2 Department of Pediatric Hematology and Oncology, Charles University Prague, 2nd Medical School, Czech Republic; 3 Institute of Pharmaceutical Biology/DCAL, University of Frankfurt, Frankfurt/Main, Germany; 4 Department of Pediatrics, Charles University Prague, 3rd Medical School, Czech Republic

## Abstract

**Background:**

While there is enough convincing evidence in childhood acute lymphoblastic leukemia (ALL), the data on the pre-natal origin in childhood acute myeloid leukemia (AML) are less comprehensive. Our study aimed to screen Guthrie cards (neonatal blood spots) of non-infant childhood AML and ALL patients for the presence of their respective leukemic markers.

**Methods:**

We analysed Guthrie cards of 12 ALL patients aged 2–6 years using immunoglobulin (Ig) and T-cell receptor (TCR) gene rearrangements (n = 15) and/or intronic breakpoints of TEL/AML1 fusion gene (n = 3). In AML patients (n = 13, age 1–14 years) PML/RARalpha (n = 4), CBFbeta/MYH11 (n = 3), AML1/ETO (n = 2), MLL/AF6 (n = 1), MLL/AF9 (n = 1) and MLL/AF10 (n = 1) fusion genes and/or internal tandem duplication of FLT3 gene (FLT3/ITD) (n = 2) were used as clonotypic markers. Assay sensitivity determined using serial dilutions of patient DNA into the DNA of a healthy donor allowed us to detect the pre-leukemic clone in Guthrie card providing 1–3 positive cells were present in the neonatal blood spot.

**Results:**

In 3 patients with ALL (25%) we reproducibly detected their leukemic markers (Ig/TCR n = 2; TEL/AML1 n = 1) in the Guthrie card. We did not find patient-specific molecular markers in any patient with AML.

**Conclusion:**

In the largest cohort examined so far we used identical approach for the backtracking of non-infant childhood ALL and AML. Our data suggest that either the prenatal origin of AML is less frequent or the load of pre-leukemic cells is significantly lower at birth in AML compared to ALL cases.

## Background

Acute lymphoblastic leukemia (ALL) is the most common type of pediatric cancer while acute myeloid leukemia (AML) occurs approximately 5 times less frequently in children. There is evidence that some childhood acute leukemias originate in utero. Indirect proof of the pre-natal origin has been given by the cases of monozygotic twins with genotype-identical leukemia [1]. Direct evidence of the prenatal origin of ALL was demonstrated in 1997. Gale et al. backtracked MLL/AF4 fusion gene, a leukemo-specific clonal marker, to neonatal dried blood spots on filter-paper (Guthrie cards) of 3 children who developed ALL at the ages of 5 months to 2 years [2]. Guthrie cards positive for the TEL/AML1 (ETV6/RUNX1) were found in more than 10 ALL patients with this fusion gene [3-6].

Clonal rearrangements of immunoglobulin (Ig) and T-cell receptor (TCR) genes are found in >90% of childhood ALL and can be used for retrospective analysis of cases without detectable chromosomal translocations. Identical rearrangements of Ig/TCR genes in twins and triplets with concordant leukemia [7-9] and their presence in Guthrie cards of patients who developed leukemia [10-12] provide satisfactory evidence of prenatal origin of ALL.

The availability of clonal markers in AML is lower. Approximately 12 % of childhood AML cases bear the translocation between chromosomes 8 and 21 resulting in AML1/ETO (RUNX1/CBFA2T1) fusion gene. Translocation t(15;17) with PML/RARalpha fusion occurs in ~6% and t(16;16) or inv(16) with chimera CBFbeta/MYH11 is found with similar frequency among children with AML [13]. Internal tandem duplications of FLT3 gene (FLT3/ITD) are always in frame but vary in length. FLT3/ITD is associated with poor prognosis and occurs in ~17% of children with AML [14].

There are only few studies concerning in utero origin of AML. Twins studies with AML patients indicate that both infant [15,16] and non-infant [17] acute myeloid leukemia can be initiated in utero. The AML1/ETO fusion gene was detected in neonatal blood spots in 5/10 t(8;21) positive patients [18]. Apart from this study, only 3 single cases of AML with positive Guthrie card were reported: for PML/RARalpha, CBFbeta/MYH11 [19] and MLL/AF10 fusion genes [20], respectively.

Our study aimed to screen Guthrie cards in larger cohort of non-infant childhood AML and ALL patients for the presence of their respective leukemic markers. Clone-specific markers used for the screening included PML/RARalpha, AML1/ETO and CBFbeta/MYH11 fusion genes, chimeric products with the MLL gene and for the first time also the FLT3/ITD in patients with AML. In ALL cases we used TEL/AML1 fusion gene and Ig/TCR gene rearrangements. These molecular markers represent suitable targets for backtracking of leukemic cells in newborn material, because they are clonotypic and their incidence is ~40% and >90% in AML and ALL, respectively. To our knowledge, this is the largest study analyzing prenatal origin of childhood leukemias presented so far.

## Methods

### Patients and controls

We studied the leukemic bone marrow cells at diagnosis or relapse together with respective Guthrie cards of 13 pediatric patients with AML and 12 with ALL after obtaining informed consent from the patients' parents. The study was in compliance with the Helsinky Declaration and was approved by the Ethical Committee of University Hospital Prague – Motol and by the board of the Czech Pediatric Hematology Working Group (CPH). All children were diagnosed and treated in one of the CPH centers. Guthrie cards were obtained from the central repositories in the Czech Republic. Guthrie cards from age-matched healthy children were used as controls for Guthrie cards analyses. Mononuclear cells from healthy donors were used as negative controls for dilution experiments.

### Preparation of DNA

DNA from patients' and donors' cells was isolated by QIAamp DNA Blood Mini Kit (Qiagen GmbH, Hilden, Germany). Alternatively, an intact, high molecular weight DNA necessary for amplification of large fragments was isolated by the salting out protocol [21].

### Determination of leukemia specific markers

Immunoglobulin and T cell receptor gene rearrangements were identified as described previously [22,23]. Clonality of the PCR products was determined by heteroduplex analysis [24]. Monoclonal PCR products were cut from the gel, reamplified with the same set of primers, purified by QIAquick PCR Purification Kit (QIAGEN, Hilden, Germany) and sequenced. FLT3/ITD were amplified by genomic DNA PCR using primers 11F (5'-GCA ATT TAG GTA TGA AAG CCA GC-3') and 12R (5'-CTT TCA GCA TTT TGA GGG CAA CC -3') as described elsewhere [25]. PCR products were resolved on a 2% agarose gel stained with ethidium bromide. Products bearing the duplication were cut out, purified with Qiagen Gel Extraction Kit (QIAGEN, Hilden, Germany) and sequenced. PML/RARalpha and CBFbeta/MYH11 intronic (DNA) breakpoints were found using multiplex long-distance polymerase chain reaction (LD-PCR). We used 10 multiplex PCR reactions for PML/RARalpha with 2 PML primers in intron 3 or 2 PML primers in intron 6 in combination with one of the 5 RARalpha primers. For each CBFbeta/MYH11, six individual PCR reactions were set up using 6 different CBFbeta primers in combination with single MYH11 primer [19]. We amplified AML1/ETO fusion breakpoint by 18 multiplex LD-PCR reactions. Nine ETO primers were combined with two pairs of AML1 primers [18]. For each TEL/AML1 patient, 30 individual PCR reactions were set up for identification of genomic fusion sequence. Primers TEL6B or TEL8B were used in combination with one of the 15 AML1 primers [26].

AML1/ETO, TEL/AML1, PML/RARalpha and CBFbeta/MYH11 LD-PCR reactions were performed using eLONGASE DNA polymerase (Invitrogen Life Technologies, Carlsbad, CA) with high molecular DNA as a template. In all LD-PCR reactions 1,7 mM MgSO_4 _was used and products were visualized on a 1,5 % agarose gel stained with ethidium bromide. Positive bands were excised from gel and purified by QIAEX II Gel Extraction Kit (QIAGEN GmbH, Hilden, Germany).

All MLL fusions sequences were established at the DCAL in Frankfurt. A long-distance inverse PCR method was used to determine the genomic fusion breakpoints [27].

Sequencing was performed in the ABI PRISM^® ^310 Genetic Analyzer with BigDye™ Primer v3.0 Sequencing Kit (Applied Biosystems, Foster City, CA). Sequencing data analyses were performed using Chromas version 1.5 software (TECHNELYSIUM, Queensland, Australia). The BLAST search was used to identify clonal Ig/TCR rearrangements, FLT3/ITD and genomic breakpoints of fusion genes (NCBI, Bethesda, MD, USA).

### Guthrie card PCR

DNA from Guthrie cards was isolated using QIAamp DNA Blood Micro Kit (Qiagen GmbH, Hilden, Germany) or by INSTAGENE™ DRY BLOOD kit (Bio-Rad, Hercules, CA, USA). Alternatively, in some cases 1/8 of Guthrie card was washed twice in 1 ml water (30 min each wash) to remove PCR inhibitors [28], air-dried and used directly in PCR. Presence and quality of DNA was assured by amplification of the beta-globin housekeeping gene.

We designed primers for nested or semi-nested PCR to amplify the patient-specific breakpoint (TEL/AML1, AML1/ETO, PML/RARalpha, CBFbeta/MYH11 and MLL rearrangements) or junctional region (Ig/TCR rearrangements). Allele specific oligonucleotide (ASO) primers were designed across the N specific segments (V-N-D, D-N-J, V-N-J or V-N-Kde) of Ig/TCR rearrangements. In samples with FLT3/ITD the 5' end primers for first and second round annealed to the junction between the wild-type sequence and the ITD sequence. The 3' end primers annealed to the wild type FLT3. Primers were designed using the VECTOR NTI 8 Suite Software (INFORMAX, Bethesda, MD, USA).

We established a PCR assay for each clonotypic sequence prior to the analysis of the corresponding Guthrie card. Assay specificity was determined using serial dilutions of patients' diagnostic DNA sample into the DNA of a healthy donor. Guthrie card PCRs were performed in a separate room in a separate PCR box and only highly diluted diagnostic samples (10^-4^-10^-5^) were used as positive controls in Guthrie card experiments. Also all other common anti-contamination procedures were followed to avoid any potential risk of contamination.

All positive products from Guthrie card experiments were sequenced to confirm the specificity of the product.

## Results

### Sensitivity of the experiments

Each PCR system for the Guthrie card analysis was optimized using different annealing temperatures, different MgCl_2 _concentrations and/or different primer concentrations before we performed the Guthrie card PCR. Desired sensitivity was > = 10^-4 ^(i.e. 1 cell from diagnostic sample among 10 000 other cells). We reached this sensitivity in all systems and in all experiments except for 2 AML patients (AML13 and AML8) who presented with a low blast percentage (5.2 and 30%, respectively) at diagnosis. In these patients, the sensitivity of some experiments (10 out of 24) was only 10^-3^.

The beta-globin control gene was amplified in all Guthrie cards. Quantitative PCR analysis of beta-globin content in 5 randomly selected samples demonstrated that amount of DNA extracted from 1 blood spot is 295 – 815 ng (median 580 ng).

### Targets, clonal-specific PCR and Guthrie card analysis

The characteristics of patients and results of the analyses are summarized in Table 1 (ALL patients) and Table 2 (AML patients). In our group of 12 ALL patients we detected 15 Ig/TCR rearrangements by heteroduplex analysis. In 3 patients we sequenced the TEL/AML1 genomic breakpoint by LD-PCR. We designed ASO primers for Ig/TCR rearrangements and specific primers amplifying the TEL/AML1 intronic breakpoint region for patients with the fusion gene. In one patient we screened the Guthrie card for both Ig/TCR and TEL/AML1. In AML patients FLT3/ITD and/or genomic breakpoints of different fusion genes (PML/RARalpha, CBFbeta/MYH11, AML1/ETO, MLL/AF6, MLL/AF9, and MLL/AF10) were used as clonal markers. In one patient from our AML cohort the PML/RARalpha fusion gene and also the FLT3/ITD were present in diagnostic sample and tested in Guthrie card.

In three patients with ALL (= 25%) we reproducibly detected identical clonotypic marker in the presentation sample and in the neonatal blood spot on the Guthrie card. The first patient harbored two independent rearrangements: TCR-delta Vd2/Dd3 and IgH VH3/JH5 and both were detectable in Guthrie card. In the second patient two PCR systems were optimized with identical sensitivity: Ig-kappa Vk1/Kde and TCRgamma VgI/Jg1.3–2.3. However, only the latter detected leukemic cells in the Guthrie card. The third patient had the diagnostic TEL/AML1 intronic fusion present in his Guthrie card. Overall, we detected positive Guthrie card in 3/15 (= 20%) of Ig/TCR markers in and 1/3 TEL/AML1 positive leukemias. We did not find patient-specific molecular markers in any Guthrie card of patients with AML.

## Discussion

We examined neonatal blood spots for the presence of clonotypic markers of (pre-)leukemic cells in the cohort of 25 patients with acute leukemia using a sensitive PCR technique.

We confirmed the results of previous studies on prenatal origin of ALL based on the same approach. In our cohort, the presence of clonotypic marker of the leukemic cells was backtracked in 3/12 ALL patients (25%). The data on the prenatal origin of AML are less comprehensive. A convincing study on AML backtracking in AML1/ETO patients was published by Wiemels et al. [18] with 5/10 patients positive. Other subtypes of non-infant childhood AML were examined rarely. There are studies with single positive Guthrie cards for MLL/AF10, PML/RARalpha and CBFbeta/MYH11 fusion genes, respectively [19,20], and to our knowledge no other cases of non-infant childhood AML were ever confirmed to be of pre-natal origin using this approach. It is more demanding to perform a large Guthrie card study on AML patients compared to ALL – the diagnosis is about five times less frequent and there is no universal marker for the genetic screening in AML cases (unlike the Ig/TCR rearrangements in ALL). In our study we were able to collect Guthrie cards from 13 AML children with a suitable molecular marker – we found intronic breakpoints in 12 patients with 6 different fusion genes and, moreover, we used for the first time FLT3/ITD for the analysis in two patients. Three different fusion genes involving MLL locus were used in our study (MLL/AF6, MLL/AF9 and MLL/AF10). MLL translocations are closely linked to congenital and infant leukemias. However, to our knowledge only three non-infant patients with MLL rearrangement detected in Guthrie card has been presented so far. The case report of MLL/AF10 positive AML mentioned above [20] and two MLL/AF4 positive patients with ALL diagnosed at 2 and 6 years, respectively [2,4]. In our study, the youngest AML patient (case AML9 with MLL/AF6 fusion gene) was 18 months old at diagnosis.

The limitation of the Guthrie card approach is the sensitivity of the assay. A segment (usually 1/8) of a blood spot analyzed in one PCR reaction contains only ~30 000 nucleated cells and the DNA on the spot can be of a poor quality (depending on the storage conditions and time). To estimate a yield of our DNA isolation from Guthrie cards we performed beta-globin housekeeping gene quantitative PCR analysis in some samples. Providing the DNA content of one cell is ~6 pg and there is approximately 200 000 – 300 000 nucleated cells in one spot our results show that we were able to extract ~20–60% of the DNA theoretically originally present on the Guthrie card. To our knowledge this type of analysis has never been shown and taking into account the storage time and conditions we believe the result is very satisfactory.

We aimed to optimize the PCR to reach the sensitivity of at least 10^-4 ^in all patients and, moreover, we designed the PCR so that the 1^st ^round amplicon is shorter than 150–200 base pairs enabling us to successfully analyze also potentially highly fragmented DNA on the Guthrie card. To determine the sensitivity of each target we used dilutions of DNA from the diagnostic sample into DNA of healthy volunteer in our dilution series. Although the optimalization of such experiments is more laborious compared to reactions using dilution into water (there is higher possibility of non-specific amplifications from the volunteer DNA) we believe this approach better reflects the real situation, offers more reliable results and should be used in all backtracking studies. We fulfilled the assessed sensitivity criteria in all targets except for two patients (AML8 and AML13) where only some of the replicates of the 10^-4 ^dilution were positive and the sensitivity was determined as 10^-3^-10^-4^. However, both these two patients had low blast count in the diagnostic sample (that serves as a 10^0 ^dilution) and thus the reached sensitivity in fact is 3 positive cells/10^-4^-10^-5 ^cells and 5 positive cells/10^-5^-10^-6 ^cells in patients AML8 and AML13, respectively. Therefore, our approach allowed us to detect the pre-leukemic clone in all samples providing 1–3 positive cells were present in the analyzed sample.

On one hand, the highest possible specificity of the analysis is crucial, however, on the other hand, the approach used in the study requires an extreme caution in terms of possible contamination. Once a clonal marker from the diagnostic sample is amplified by PCR and used in Guthrie card experiments as a positive control, threat of contamination can not be excluded. Moreover, it is impossible to distinguish a sporadic contamination from real positivity. We applied very strict anti-contamination precautions. The Guthrie card experiments were performed in a separate room and only highly diluted diagnostic samples (10^-4^-10^-5^) were used as positive controls. During the study we experienced no contamination in any of the negative-control samples.

The results from the ALL cohort confirm that our approach is appropriate both for the Ig/TCR targets (3/15 positive) and for the fusion gene analysis (1/3 positive).

Previously, Wiemels et al reported 5 out of 10 t(8;21) childhood AML cases with fusion gene positive Guthrie cards [18]. Only single examples of t(15;17), inv(16) and MLL-AF10 with fusion gene positive Guthrie cards have been reported [19,20]. As the present study included only two cases of AML1-ETO positive leukemia, it is not possible to say that this occurs prenatally at a higher frequency.

Young age at diagnosis is probably not a crucial prerequisite for the pre-natal origin of childhood acute leukemia as cases with a long latency to leukemia onset were described [1,4,7,18,19,29]. However, the fact that the ALL patients in our study were significantly younger at diagnosis compared to AML patients (median 3.5 and 6 years, respectively) should be mentioned here.

The negative result in Guthrie card analysis can be caused at least by three different reasons (providing the sensitivity of detection is adequate) – the leukemia does not originate prenatally, the pre-leukemic clone at birth is too small to be present at Guthrie card spot or the marker used for the backtracking is not the initiating or early event in leukemogenesis. The latter is probably the case with the FLT3/ITD. This abnormality was never tested before as a marker of leukemic cells in Guthrie cards. The positivity of this abnormality in neonatal blood is rather unlikely as the latest data indicate that this aberration has probably a post-natal origin [30,31]. And indeed, we did not detect the FLT3/ITD in any of the Guthrie cards tested.

The sensitive patient-specific PCR targeted either to the FLT3/ITD breakpoint or to the intronic fusion of the various chimeric genes provide potentially a very useful tool not only for backtracking of leukemia but also for the minimal residual disease monitoring in almost 50% of children with AML, where common molecular marker is unavailable (unlike Ig/TCR rearrangements in ALL).

In our study on childhood non-infant acute leukemias we confirmed that the prenatal origin is common in ALL patients (25% in our cohort) while all neonatal blood spots in our AML cohort were negative. Although we found all of the Guthrie cards from AML patients negative, we do not think that our results completely contradict the previous reports with positive neonatal blood spots of AML patients [18-20]. However, we feel that with the exception of AML1/ETO fusion gene, where the prenatal origin has been proven reliably, reports of prenatal origin in non-infant childhood AML are isolated single cases. These should be confirmed by other studies or reports as a false-positivity can not be ruled out completely with the approach used. For example the E2A/PBX1 fusion gene was found to be "weakly" positive in Guthrie cards of 2 out of 15 patients tested, and, nevertheless, the data on E2A/PBX1 are interpreted as being suggestive of a post-natal origin of this fusion gene [32].

## Conclusion

The results of our study using the same approach for both ALL and AML clearly indicate that either the prenatal origin of AML is less frequent or the load of pre-leukemic cells is significantly lower at birth in AML compared to ALL cases.

## Competing interests

The author(s) declare that they have no competing interests.

## Authors' contributions

TB, JM and KM carried out the molecular genetic experiments with Guthrie cards and controls. CM, BS and RM sequenced the genomic breakpoints of fusion genes. FV traced the Guthrie cards and helped with data analysis. JT and JS participated in the design of the study and helped to draft the manuscript. JZ conceived of the study, and participated in its design and coordination and helped to draft the manuscript.

All authors read and approved the final manuscript.

## Pre-publication history

The pre-publication history for this paper can be accessed here:



## Figures and Tables

**Table 1:** Results of Guthrie cards screening in patients with ALL

*Patient*	*Molecular marker*	*Detection sensitivity*	**% blasts at diagnosis**	**Age at diagnosis**	*Guthrie card*
ALL1	Vd2	10^-4^	N.A.	2 y 8 mo	negative
	VH3	10^-5^			negative
ALL2	VH3	10^-4^	50%	3 y 3 mo	negative
	TEL/AML1	10^-4^-10^-5^			negative
ALL3	VgI	10^-5^	>55%	4 y 5 mo	positive
	VkIA	10^-4^			negative
ALL4	Vd2	10^-5^	N.A.	3 y 6 mo	positive
	VH3	10^-4^			positive
ALL5	VH3	10^-5^	70%	4 y	negative
ALL6	VH3	10^-4^	84%	1 y 8 mo	negative
	VH1	10^-5^			negative
ALL7	VH1	10^-5^	83%	3 y 3 mo	negative
ALL8	VH3	10^-4^	79%	2 y 6 mo	negative
ALL9	VH3	10^-4^	89%	2 y 3 mo	negative
	VH4	10^-5^			negative
ALL10	VgI	10^-4^	>30%	3 y 9 mo	negative
ALL11	TEL/AML1	10^-4^-10^-5^	80%	5 y 7 mo	negative
ALL12	TEL/AML1	10^-5^	77%	3 y 6 mo	positive

**Table 2:** Results of Guthrie cards screening in patients with AML

*Patient*	*Molecular marker*	*Detection sensitivity*	**% blasts at diagnosis**	**Age at diagnosis**	*Guthrie card*
AML1	FLT3/ITD	10^-5^	87%	2 y 9 mo	negative
AML2	FLT3/ITD	10^-5^	70%	8 y 9 mo	negative
	PML/RARalpha	10^-4^-10^-5^			negative
AML3	PML/RARalpha	10^-4^-10^-5^	>80%	13 y 9 mo	negative
AML4	PML/RARalpha	10^-4^	55%	8 y 7 mo	negative
AML5	PML/RARalpha	10^-4^-10^-5^	64%	7 y 4 mo	negative
AML6	CBFbeta/MYH11	10^-4^-10^-5^	82%	5 y 8 mo	negative
AML7	CBFbeta/MYH11	10^-4^	70%	6 y 3 mo	negative
AML8	CBFbeta/MYH11	10^-3^-10^-4^	30%	2 y 6 mo	negative
AML9	MLL/AF6	10^-4^-10^-5^	73%	1 y 5 mo	negative
AML10	MLL/AF9	10^-4^-10^-5^	70%	1 y 7 mo	negative
AML11	MLL/AF10	10^-4^	78%	5 y 4 mo	negative
AML12	AML1/ETO	10^-4^-10^-5^	40%	9 y 4 mo	negative
AML13	AML1/ETO	10^-3^-10^-4^	5,2%	14 y 8 mo	negative
